# A Method for Fast Assessment of OP/CB Exposure in the Japanese Quail (*Coturnix coturnix japonica*) Using Combined Esterases Enzyme Activity as Biomarkers

**DOI:** 10.1155/2014/812302

**Published:** 2014-01-09

**Authors:** Kasim Sakran Abass

**Affiliations:** Department of Basic Nursing Sciences, College of Nursing, University of Kirkuk, Kirkuk, Iraq

## Abstract

The aims of this study were to investigate the presence of different esterase activities in plasma and liver for Japanese quail and to combine determination of both carboxylesterase and cholinesterase as biochemical biomarker in order to identify the effects of carbamate and organophosphate compounds exposure. Carboxylesterase exhibits larger sensitivity to carbamate and organophosphate compounds than to cholinesterase and is present at higher levels. This permitted nature and distribution of carboxylesterase or cholinesterase to be measured. One predominant toxicological form of enzyme level constant in its patterns of motivation and inhibition with cholinesterase was identified in plasma with an apparent Michaelis constant for butyrylthiocholine iodide of 0.394 mM. Carboxylesterase activity in liver was considered by its preferential hydrolysis of the S-phenyl thioacetate. A concentration dependent decrease of carboxylesterase and cholinesterase has demonstrated during *in vitro* incubation of malathion, parathion, and trichlorfon in the range 0.125–2 mM, while with methomyl was in the range 0.25–4 mM. When quail (*n* = 15) was exposed orally for 48 h to concentrations of carbamate or organophosphate compounds of 3–200 mg/kg, the percentage inhibition of cholinesterase was in each case larger than that of carboxylesterase and reached statistical significance (*P* < 0.05) at lower concentrations.

## 1. Introduction

Enzymes that preferentially catalyse the hydrolysis of ester bonds are classified as esterase (EC 3.1) and generally classified into two subgroups, carboxylesterase (CbE; EC 3.1.1.1) and cholinesterase (ChE; EC 3.1.1.7), depending on their substrate specificity and behaviour towards some inhibitors [1, 2]. There are many esterase decreases among carbamate (CB) and organophosphate (OP) compounds, which are the most important in the agriculture and veterinary medicine to control insect invasion; these compounds exert their toxic effects through inhibiting esterase enzyme activities [3–5]. CB and OP compounds exert acute toxicity by inhibition ChE, a serine hydrolase found in neuromuscular junctions of birds (connects the nervous system to the muscular system via synapses) as well as in peripheral and central cholinergic synapses [6, 7]. This leads to susceptible types in overaccumulation of the excitatory neurotransmitter acetylcholine and subsequent hyperpolarisation of the postsynaptic membrane. Determination of CbE and ChE enzyme activities levels is a valuable, dose dependent means of monitoring exposure to CB and OP pesticides in animal models and other vertebrate species [8].

The ChE exhibit toxicity of distributions and their molecular polymorphism and biological roles differ among types [9]. Therefore, the degree of decreases associated with toxicity is highly variable [10]. Generally, the enzyme activity may be modulated by seasonal and nutritional variables [8] or by chronic toxic exposures which can lead to higher levels of enzyme activity [6]. Quails are found widely in coastal and shallow temperate areas as large beds in both natural waters and brackish estuaries where agricultural excess at its peak. Although the occurrence of ChE enzyme activity in blood plasma samples was first described many years ago [11], there have been relatively few papers published studies of the use of CbE or ChE enzyme activities as a biomarker of OP compounds exposure in quail [12]. Additionally, their lethal action on ChE with CB and OP compounds may also affect other classes of serine hydrolase including CbE that are naturally present in liver [13]. The CbE enzyme hydrolyses a varied range of exogenous and endogenous esters [14].

Their liver distribution and exact biological role is often unidentified and differs among species, but they may supposedly be essential in the hydrolytic detoxification of some OP pesticides and play an extra protecting role as alternate locations of OP binding and phosphorylation [15]. The CbE enzyme activity in quail exhibits higher sensitivity to OP pesticides than ChE [1]; also its measurable enzyme activity is greater [16]. Consequently, having made it possible to evaluate combined monitoring of CbE and ChE activities may provide a more valuable indication of CB and OP compounds exposure in quail models than the determination of ChE enzyme activity alone. The distribution and characteristics of CbE and ChE enzyme activities then measured and the sensitivity of the enzyme activities to inhibition both *in vitro* and *in vivo* to CB and OP compounds were evaluated. At the end, the objectives of this paper were (a) to investigate the presence of different esterase enzyme activities in blood plasma and liver for quail; (b) to use the esterase enzyme activities as biochemical biomarker for the evaluation of exposure to CB and OP pesticides; and (c) to combine determination of both CbE and ChE enzyme activities in the quail in order to identify the effects of CB and OP compounds exposure.

## 2. Materials and Methods

### 2.1. Materials

CbE substrate, S-phenyl thioacetate (PSA), 98% purity (chemical name: thiophenyl acetate, molecular structure: CH_3_COSC_6_H_5_, molecular mass: 152.2 g mol^−1^); ChE substrates, acetylthiocholine iodide (AcTChI), 98% purity (chemical name: (2-mercaptoethyl) trimethylammonium iodide acetate, molecular structure: CH_3_COSCH_2_CH_2_N(CH_3_)_3_I, molecular mass: 289.2 g mol^−1^); S-butyrylthiocholine iodide (BuTChI), 98% purity (chemical name: (2-mercaptoethyl) trimethylammonium iodide butyrate, molecular structure: (CH_3_)_3_N(I)CH_2_CH_2_SCOCH_2_CH_2_CH_3_, molecular mass: 317.2 g mol^−1^); propionylthiocholine iodide (PrTChI), (chemical name: (2-mercaptoethyl) trimethylammonium iodide propionate, molecular structure: C_8_H_18_NOSI, molecular mass: 303.2 g mol^−1^); Ellman reagent, 5,5′-dithiobis(2-nitrobenzoic acid) (DTNB) (chemical name:3-carboxy-4-ntrophenyl disulfide,molecular structure: [SC_6_H_3_(NO_2_)CO_2_H]_2_, molecular mass: 396.35 g mol^−1^); OP compounds, parathion (chemical name: O,O-diethyl O-(4-nitrophenyl) phosphorothioate, molecular structure: C_10_H_14_NO_5_PS, molecular mass: 291.26 g mol^−1^) and trichlorfon (chemical name: dimethyl (2,2,2-trichloro-1-hydroxyethyl) phosphonate, molecular structure: C_4_H_8_C_13_O_4_P, molecular mass: 257.44 g mol^−1^); CB compound, methomyl (chemical name: S-methyl-N((methylcarbamoyl)oxy) thioacetimidate, molecular structure: C_5_H_10_N_2_O_2_S, molecular mass: 162.21 g mol^−1^) were supplied by the Sigma Chemical Company. OP (malathion) (chemical name: S-1,2-bis(ethoxycarbonyl) ethyl O,O-dimethyl phosphorodithioate, molecular structure: C_10_H_19_O_6_PS_2_, molecular mass: 330.36 g mol^−1^) was obtained from G.L. Industries (E) Ltd., Guwahati, India. All other reagents and solvents used in this paper were of analytical grade.

### 2.2. Animals

Two strains of Japanese quail (*Coturnix coturnix japonica*), namely, brown and white models, and they were maintained in batches of 3–15 quails in cages with dimensions of 75 × 75 × 75 cm in a room with constant lighting at a temperature of 25–35°C and relative humidity was between 45 and 50%, which was controlled by electric heaters. The floor litter consisted of wood shavings; water and feed were available ad libitum. The age of the quail used in the experiments ranged between 4 and 8 weeks. The present study has conducted and approved at the College of Agriculture at the University of Tikrit (Iraq). All the experiments were observed with institutional regulations addressing animal use, and good attention and care were given to the quails used in this work. During sample collection from the birds, it was ensured that there was no possibility of the introduction of pesticide compounds from the skin of the birds. As noted by Fairbrother et al. [17], this can be a source of contamination by anti-CbE or ChE.

### 2.3. Sample Preparation

To obtain plasma, 1 mL blood samples were added to anticoagulant (heparin, 3.7 mg, final concentration 0.1%) in 5 mL centrifuge tubes. Plasma was separated by centrifugation at 5000 g for 10 min. The liver was removed using a scalpel, cut into small pieces (2-3 mm^3^), and rinsed until the blood was fully removed. The liver was then placed on ice in 5 mL tubes (5 mm internal diameter) and homogenized using a mechanically driven homogenizer with phosphate buffer (0.1 M, pH 8) with a 1 : 10 ratio (w/v) of 1 part of liver tissue to 9 parts of buffer and a speed of 10000 rpm. Homogenisation required 3 min; after every one min or so of homogenization, the mixture was rested for 30 s to allow cooling. The homogenate was then centrifuged at 5000 g for 10 min and the supernatant fraction was retained and used for subsequent analyses. It was essential during tissue homogenization to ensure that (i) liver samples were fully homogeneous and that aliquots taken reflected the homogenate as a whole and (ii) that enzyme activities were not altered in the process (through heat-induced denaturation) [17].

### 2.4. Enzyme Activity Measurement

Enzyme activity was determined at room temperature 25°C by the Ellman method [18], and using thioacetate (PSA) for measuring CbE enzyme activity or by using thiocholine (e.g., AcTChI, BuTChI, or PrTChI substrate) for measuring ChE enzyme activities [19]. Subsequent combination of thioacetate or thiocholine derivatives with DTNB forms the yellow anion 5-thio-2-nitrobenzoic acid (TNB), which absorbs strongly at 410 nm [20]. Substrate solutions (final concentration 2 mM for PrTChI and PSA, while 1 mM for AcTChI and BuTChI) were prepared and used on the same day and kept on ice during use. Enzyme activities were calculated using an extinction coefficient of 13.6 mM^−1 ^cm^−1^ for TNB [21] and are expressed as units (1 U *≡* 1 *μ*mol *≡* 1000 nmol of substrate hydrolysed per min) per g wet weight of liver homogenate, while for plasma they are expressed as U/mL [2]. All measurements in this paper were carried out in duplicate.

### 2.5. *In Vitro* Exposure to Pesticide Compounds

Samples were incubated with appropriate concentrations CB or OP pesticide compounds (methomyl, malathion, parathion or trichlorfon) for 30 min at room temperature 25°C prior to the addition of substrate. Then esterases enzyme activity was measured as explained in the above section. For the measurement of half maximal inhibitory concentrations (IC_50_), samples were inhibited for 30 min at room temperature 25°C with appropriate concentration of CB or OP pesticide compounds, depending on preliminary range finding tests. Controls that were incubated with phosphate buffer pH 8.0 were included. Then the enzyme activity was determined as described above. Then the data were fitted with nonlinear regression analysis using a single exponential decay by SigmaPlot 11 (Systat software, Inc.).

### 2.6. Determination of Inhibition Rate Constants (*k*_*i*_)

For the measurement of rate constants of inhibition (*k*_*i*_), esterases enzyme activity was inhibited with 2 mM malathion, parathion, or trichlorfon concentrations or 4 mM methomyl concentration resulting in an inhibition of 83–99% of control activity. Controls that were incubated with phosphate buffer pH 8.0 were included when appropriated. Blanks were also run at each selected inhibitor for each based on the absorbance tested. Then the enzyme activity was determined as described in above. The decrease in esterases enzyme activity over different times (1–60 min) at room temperature 25°C was detected. Then the data of inhibition time courses at different times after inhibition was fitted with a single exponential decay using SigmaPlot 11. However, the percentage inhibition was calculated from the ratio between the activity of an exposed sample and unexposed controls by the following formula:

(1) %  Inhibition =Esterases  enzyme  activity  with  inhibitorEsterases  enzyme  activity  without  inhibitor×100.

### 2.7. *In Vivo* Exposure to Pesticide Compounds

The extent of inhibition of esterases enzyme activity was also assessed following *in vivo* exposure of test of quail by oral dosing (*n* = 15) were exposed to test solutions of parathion, malathion, methomyl, trichlorfon, or control (water) in cages. Hereby, final concentration of solvent did not exceed 0.09% (v/v). Samples were prepared and assayed as explained in the above section. The median lethal dose (LD_50_) values for oral doses of CB and OP compounds used in this paper for quails were (mg/kg) methomyl 24.2 [22]; parathion 6 [23]; malathion 400 [24]; and trichlorfon 22 [25]. In this experiment, three quails in each group were used and the highest sublethal acute dose; 50% of LD_50_ was used in this study and the quail tolerated the highest dose with minimal toxic symptoms. The toxic symptoms were mostly neurological.

### 2.8. Statistical Analysis

Conventional statistical methods were used to calculate the means, standard error (SE), and coefficient of variance (CV). One-way analysis of variance (ANOVA) was applied to test for any significant differences (associated probability < 0.05). The Bland-Altman method was also used to compare between two methods as described in Dewitte et al. [26]. All statistics were carried out using MiniTab statistical software version 15 (MiniTab Ltd., Coventry, UK).

## 3. Results

### 3.1. Substrate Preference and Distribution of CbE and ChE Enzyme Activity

**Figure 1:**
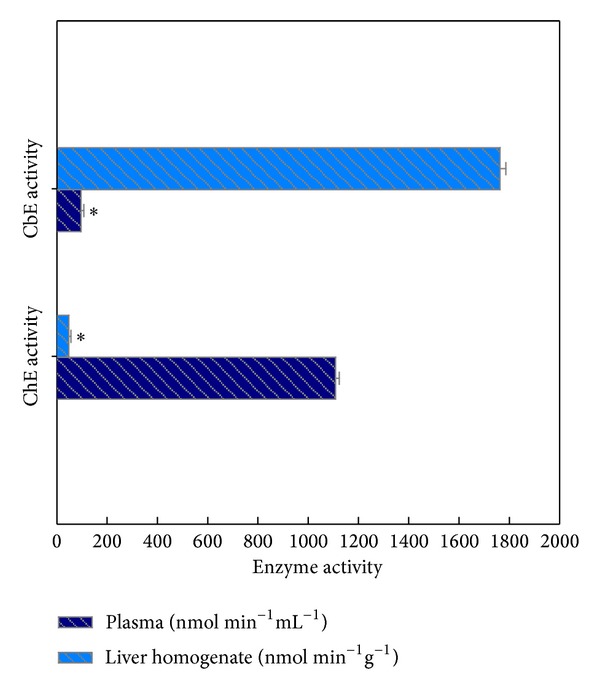
Esterase enzyme activities in blood plasma sample (nmol min^−1^ mL^−1^) and liver homogenate (nmol min^−1^ g^−1^) of quail. All values in the figure are the mean ± SE of fifteen individual quails and each is assayed in duplicate by using 2 mM of PSA as substrate for measurement of CbE enzyme or 1 mM of BuTChI as substrate for measurement of ChE enzyme. *Significant difference (analysis of variance (ANOVA), *P* < 0.05) between blood plasma sample and liver homogenate in the same enzyme (CbE or ChE).

**Figure 2:**
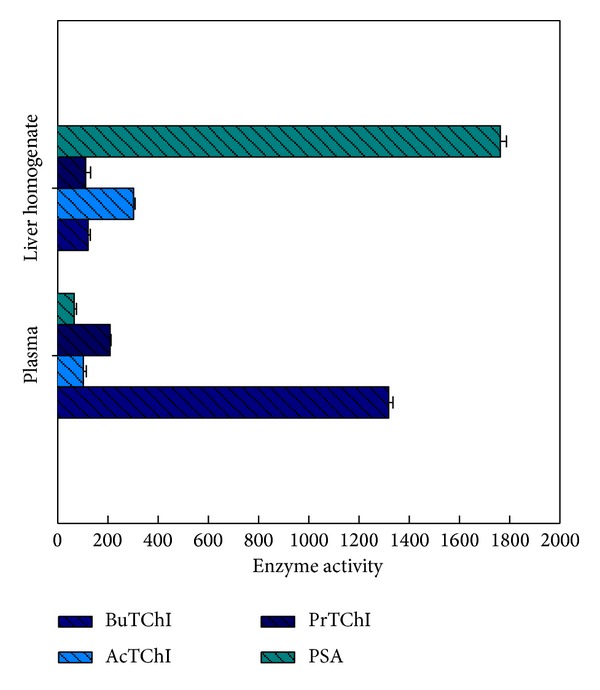
Substrate preference for blood plasma sample (nmol min^−1^ mL^−1^) and liver homogenate sample (nmol min^−1^ g^−1^) preparations. Carboxylase enzyme activity was measured in the presence of individual substrates (final concentration was 2 mM for PrTChI and PSA while 1 mM for BuTChI and AcTChI) and the maximum rate recorded. All values in the figure are the mean ± SE of fifteen individuals quail and each is assayed in duplicate.

The standard level of ChE in plasma samples was confirmed at a higher than in the liver homogenate (*P* < 0.05), whereas the reverse was true for CbE (*P* < 0.05) (Figure 1). Subsequently, ChE enzyme activity was measured highest in plasma samples (1.12 *μ*mol min^−1^ mL^−1^) as well as CbE enzyme activity was detected higher in liver homogenate (1.77 *μ*mol min^−1^ g^−1^). The limits of acknowledgment of the CbE and ChE enzyme activities were calculated by determining the reaction rate for fifteen replicates of an enzyme activity equivalent to blank (Figure 1). The limits of reaction were measured for three preference substrates for ChE enzyme in plasma which gave a sensitivity of reaction of (1321.5, 106.1, and 211.9 nmol min^−1^ mL^−1^ for BuTChI, AcTChI, and PrTChI hydrolysis, resp.) and 68.9 nmol min^−1^ mL^−1^ for PSA hydrolysis, whereas the reaction in liver homogenate gave a sensitivity of (123.8, 304.5, and 113.9 nmol min^−1^ g^−1^ for AcTChI, BuTChI, and PrTChI hydrolysis, resp.) and 1.765 *μ*mol min^−1^ g^−1^ for PSA hydrolysis (Figure 2). The mean control value for plasma was 427.1 ± 12.5 nmol min^−1^ mL^−1^ for the AcTChI, BuTChI, and PrTChI hydrolysis and representing a CV of 7.4% for the AcTChI, BuTChI, and PrTChI hydrolysis, while the mean values were 77.4 ± 7.9 nmol min^−1^ mL^−1^ for PSA hydrolysis, representing a CV of 4.1%, and the mean control value for liver homogenate 576.8 ± 15.5 nmol min^−1^ g^−1^ for the AcTChI, BuTChI, and PrTChI hydrolysis and representing a CV of 13.9% for the AcTChI, BuTChI, and PrTChI hydrolysis. While the mean values was 211.4 ± 11.3 nmol min^−1^ g^−1^ for PSA hydrolysis, representing a CV of 9.1%.

**Figure 3:**
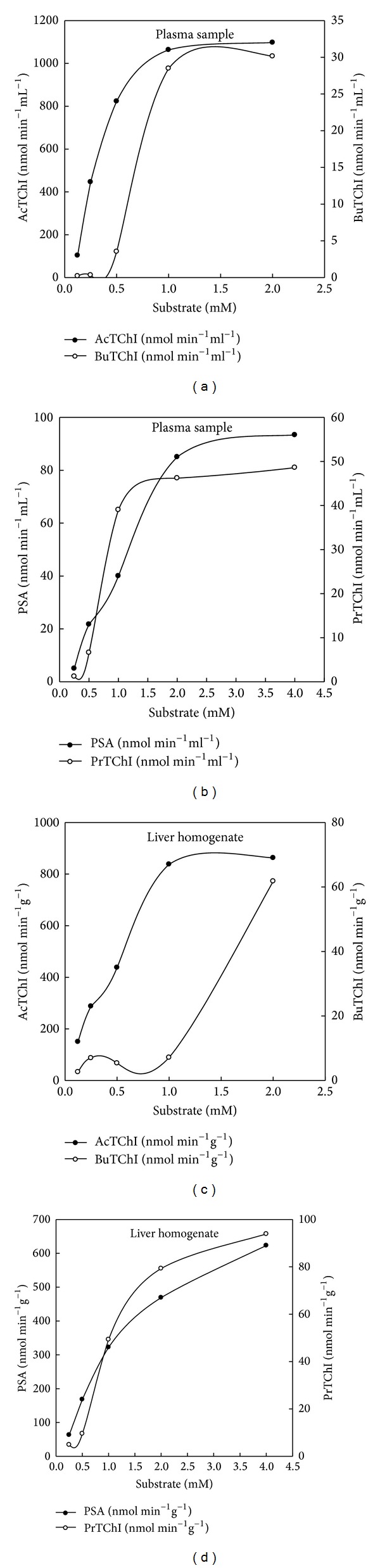
Levels of esterases enzyme (CbE or ChE) activity by using thiocholine (AcTChI, BuTChI, or PrTChI) and thioacetate (PSA) substrate (concentration was ranged from 0.125 to 2 mM for AcTChI and BuTChI while ranged from 0.25 to 4 mM for PrTChI and PSA) in blood plasma samples and liver homogenate for quails. All values in the figure are the mean of five individual quails and each is assayed in duplicate.

The substrate preference of the plasma preparation was measured by comparing rates of reaction with concentrations in the range 0.125, 0.25, 0.5, 1, and 2 mM of the substrate analogues AcTChI and BuTChI while in the range 0.25 was 0.5, 1, 2, and 4 mM for PrTChI and PSA, each of those may be favourably hydrolysed by different classes of esterase enzyme (Figures 3(a)–3(d)). AcTChI was the preferred substrate for the plasma samples (*P* < 0.05). The hydrolysis of the BuTChI and PrTChI substrates was much lower, indicating that ChE enzyme activity was the predominant enzyme activity in the plasma (Figures 3(a)–3(d)). Liver homogenate showed a preferential ability to hydrolyse PSA (*P* < 0.05) with AcTChI, BuTChI, and PrTChI hydrolysed in reducing order of preference (Figures 3(a)–3(d)). Incubation with 3 mM phenylmethanesulfonyl fluoride, a general inhibitor of CbE and ChE enzyme having active site serine residues, reduced the level of CbE and ChE enzyme activities to insignificant levels, as did denaturing the CbE and ChE by boiling the samples for 3 min. The hydrolysis of the AcTChI, BuTChI, PrTChI, and PSA was, therefore, entirely due to the effect of serine dependent CbE and ChE enzyme but did not spontaneously hydrolyse.

### 3.2. Characterisation of ChE Enzyme Activity

**Figure 4:**
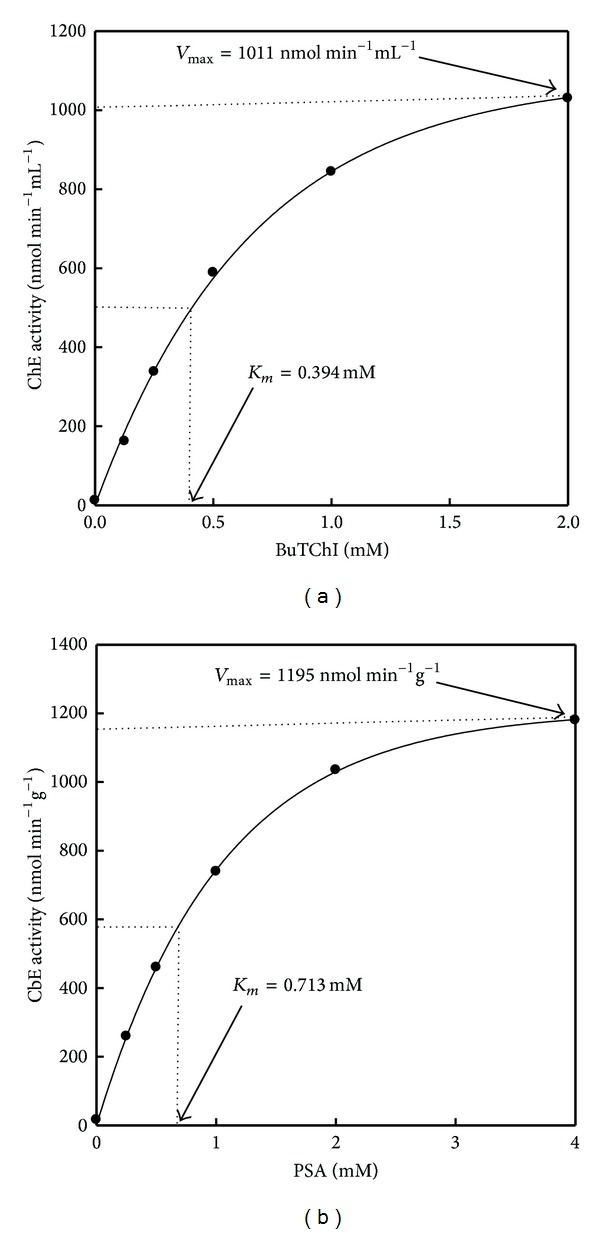
The illustrated figure is the data analysis used to obtain maximum reaction velocity (*V*_max_). The data are for quail (a) blood plasma ChE and (b) liver CbE. In each case data were fitted with nonlinear regression analysis using a single rectangular hyperbola by SigmaPlot 11.

**Figure 5:**
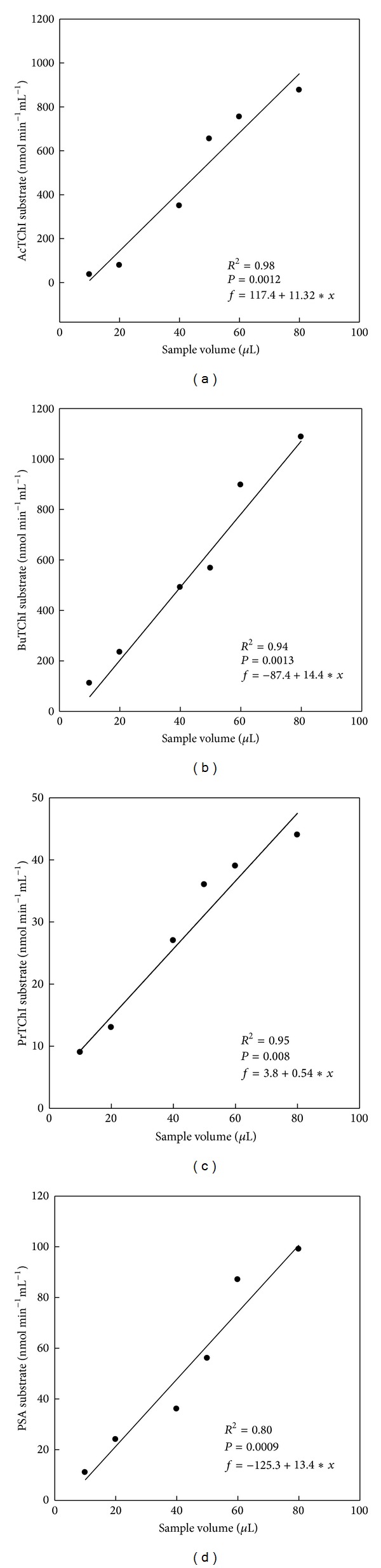
Regression analysis of blood plasma sample, between reaction rate (nmol min^−1^ mL^−1^) and blood plasma sample volume ranging of 10, 20, 40, 50, 60, and 80 *μ*L for AcTChI (a), BuTChI (b), PrTChI (c), and PSA (d). All values in the figure are the mean of three individual quails and each is assayed in duplicate.

**Figure 6:**
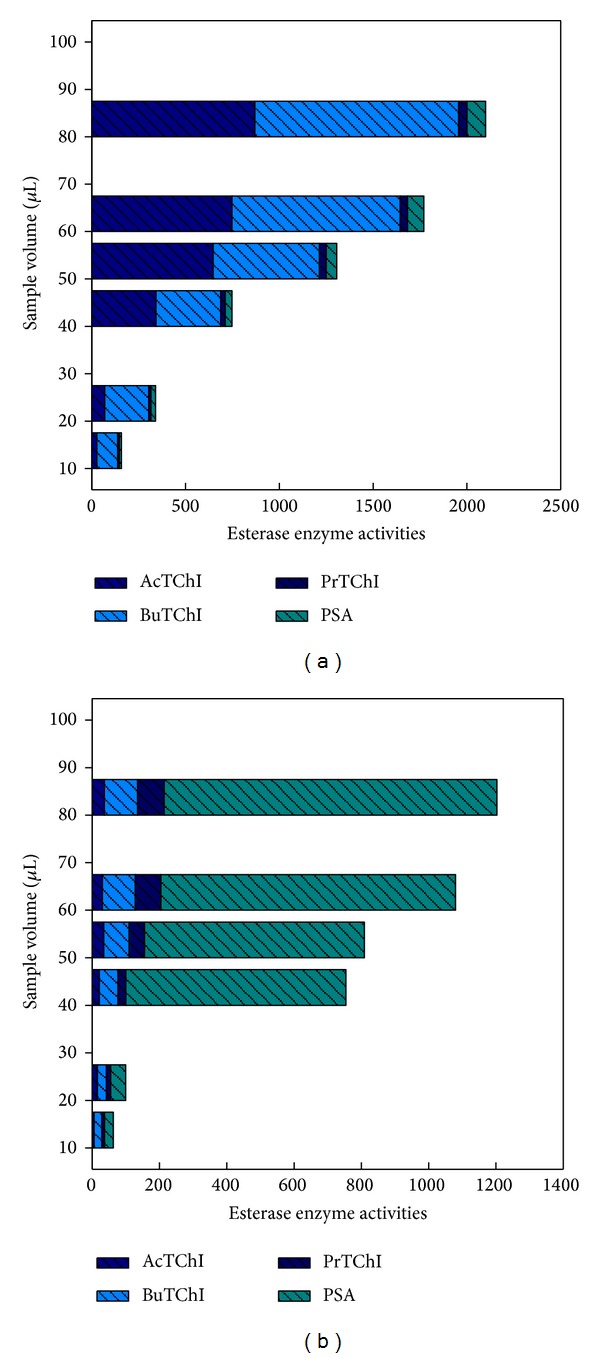
Esterase enzyme activities and sample volume ranging from 10 to 80 *μ*L for blood plasma sample (a) and liver homogenate (b). The values were obtained from Figures 5 and 8.

The concentration of BuTChI was varied to give concentrations in the range 0.125, 0.25, 0.5, 1, and 2 mM. The *K*_*m*_ value calculated using the Michaelis Menten kinetics equation was 0.394 mM. The maximum reaction velocity (*V*_max_) was achieved at 2 mM and this concentration used for consequent analysis in the information that it was not rate limiting above the 10 min time period chosen (Figure 4(a)). A distinguishing characteristic of ChE is the occurrence of substrate decreases at high concentrations and this phenomenon can be seen clearly in Figures 5(a)–5(c). A serial dilution of plasma samples was completed in buffer to confirm linear kinetics. The study of reaction rates was directly proportional to ChE concentration across the range of dilutions tested confirming that Michaelis-Menten kinetics was supported in this range (Figures 5(a)–5(d)). The sample volume subsequently chosen naturally yielded reaction rates for individual quail in the upper third of this range (50 *μ*L), allowing for the accurate recognition of inhibition. The *R* ^2^ values in blood plasma samples ranging from 10 to 80 *μ*L for AcTChI, BuTChI, PrTChI, and PSA substrates were tended to be very high in case of AcTChI activity (*R* ^2^ = 0.98, *P* = 0.0012; Figure 5(a)) and lower in case of PSA (*R* ^2^ = 0.80, *P* = 0.0009; Figure 5(d)). The mean differences in the individual samples volume reaction for plasma and liver homogenates between two enzymes are plotted and showed high level of esterases enzyme activity when using BuTChI as substrate in plasma (Figure 6(a)) whereas was a high level of esterases enzyme activity when using PSA as substrate in liver homogenate (Figure 6(b)).

### 3.3. Characterisation of CbE Enzyme Activity

**Figure 7:**
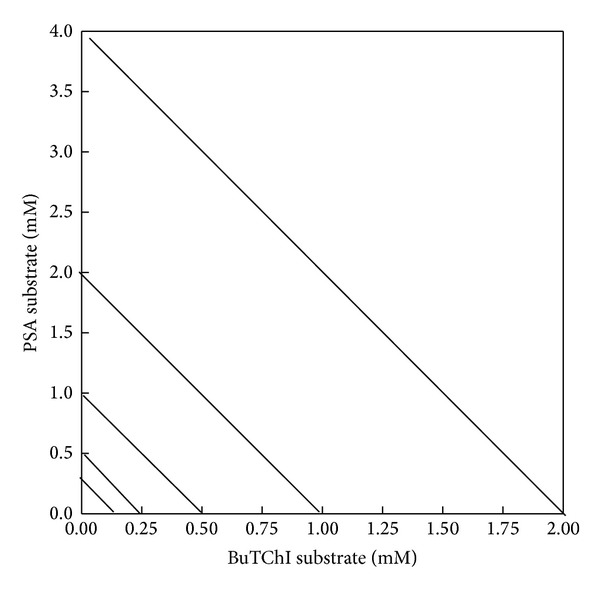
Comparison of two substrate concentrations. The lines between *y*-axis and *x*-axis are representing the relationship between the concentrations of PSA and BuTChI, respectively (mM).

**Figure 8:**
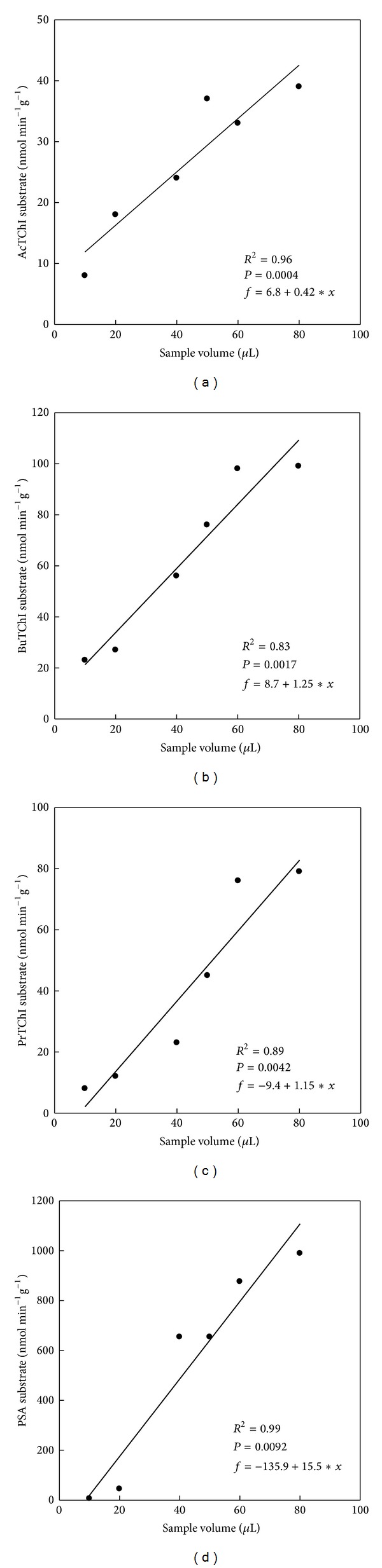
Regression analysis of liver homogenate, between liver homogenate reaction rate (nmol min^−1^ g^−1^) and sample volume range of 10, 20, 40, 50, 60, and 80 *μ*L, for AcTChI (a), BuTChI (b), PrTChI (c), and PSA (d). All values in the figure are the mean of three individual quails and each is assayed in duplicate.

**Figure 9:**
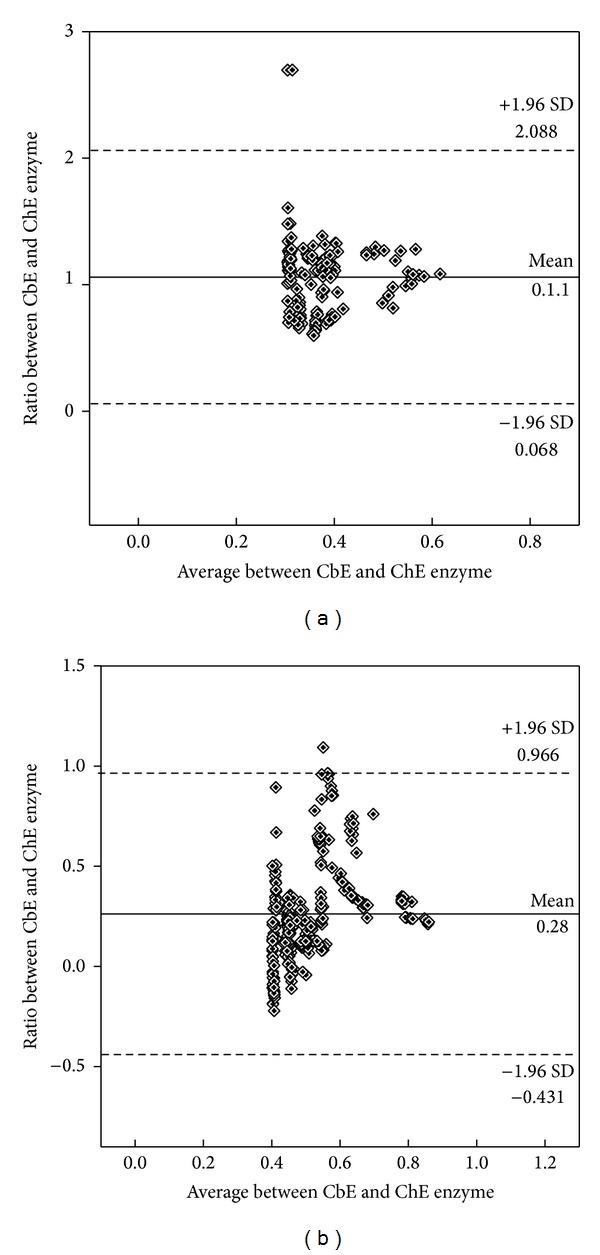
Bland and Altman plot of the ratio of CbE and ChE enzyme (plotted on the *y*-axis) versus the average of the two enzymes (*x*-axis) for AcTChI, BuTChI, PrTChI, and PSA substrates in the individual samples of blood plasma and liver homogenate for quail using (a) uninhibited enzymes and (b) inhibited enzymes with CB and OP pesticide compounds. Horizontal lines are drawn at the mean difference and at the mean difference ±1.96 SD of the differences (dashed line). If the differences within mean ±1.96 SD are clinically not important, the two enzymes cannot be used interchangeably.

A test for concentration of PSA was different to give concentrations in the range 0.25, 0.5, 1, 2, and 4 mM. An apparent *K*_*m*_ value of 0.713 mM was detected for the measured CbE enzyme activity. The *V*_max_ was achieved at 3.8 mM, and this concentration was used for consequent work (Figure 4(b)). Final substrate concentrations range from 0.25 to 4 mM for PSA and from 0.125 to 2 mM for BuTChI (Figure 7). At higher concentrations, a precipitate formed on addition of the sample, making confirmation of substrate decreases at higher doses impractical. A serial dilution of liver was completed in evaluate buffer to approve linear kinetics (Figures 8(a)–8(d)). The study of reaction rates were directly proportional to enzyme concentration across the range of dilutions tested approving that Michaelis-Menten kinetics were supported on a range. A sample volume of 50 *μ*L was also chosen for subsequent analysis which naturally gave enzyme activity values for individual quail in the upper ranges of the assay yet was low sufficient to prevent precipitation with PSA. The *R* ^2^ values in liver homogenate samples with the ranging from 10 to 80 *μ*L for AcTChI, BuTChI, PrTChI, and PSA substrates was tended to be very high in case of PSA activity (*R* ^2^ = 0.99, *P* = 0.0022; Figure 8(d)) and lower in case of BuTChI (*R* ^2^ = 0.80, *P* = 0.0017; Figure 8(b)). Lastly, the mean differences in the individual samples for plasma and liver homogenates between two enzymes inhibited are plotted by Bland and Altman plot and showed clinically important, due to the mean being found lesser than ±1.96 (Figure 9(a)).

### 3.4. *In Vitro* Inhibition of Enzyme Activity

**Figure 10:**
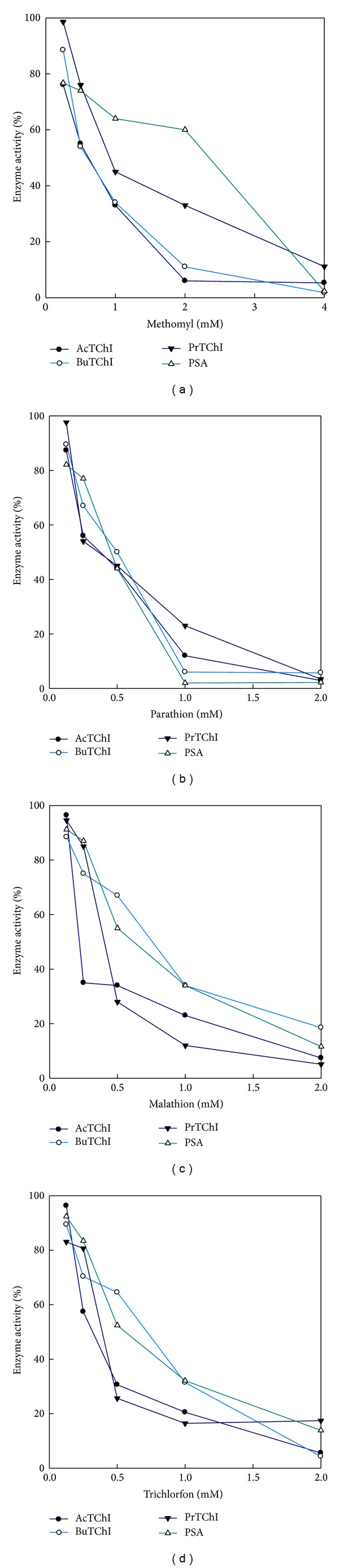
The effect of CB and OP pesticides on esterase enzyme activities in quail. The esterase activities were measured in blood plasma sample. Results are expressed as a % of the control (a) methomyl (0.25–4 mM); (b) parathion (0.125–2 mM); (c) malathion (0.125–2 mM); and (d) trichlorfon (0.125–2 mM). All values in the figure are the mean of three individual quails and each is assayed in duplicate.

The sensitivity of the CbE or ChE enzyme activity in plasma and liver homogenate samples to decreases by CB and OP pesticides was initially investigated *in vitro*. The selected compounds were the CB methomyl (0.25–4 mM) a potent inhibitor of CbE or ChE and the OP pesticides malathion (phosphorodithioate) (0.125–2 mM), parathion (phosphorothioate) (0.125–2 mM), and trichlorfon (phosphonate) (0.125–2 mM). These compounds were chosen, as they do not require metabolic activation prior to use (Figure 10). Plasma percentage inhibition of methomyl almost ranged AcTChI (5.3–76.1%), BuTChI (1.8–88.5%), PrTChI (11.1–98.6%), and PSA (2.3–76.7%) (Figure 10(a)); parathion ranged AcTChI (2.9–87.3%), BuTChI (5.7–89.5%), PrTChI (3.4–97.6%), and PSA (2.2–82.1%) (Figure 10(b)); malathion ranged AcTChI (7.4–9.6%), BuTChI (18.5–88.4%), PrTChI (5.1–94.5%), and PSA (11.6–91.2%) (Figure 10(c)); and trichlorfon ranged AcTChI (5.6–96.3%), BuTChI (4.3–89.4%), PrTChI (17.4–83.1%), and PSA (13.8–92.4%) (Figure 10(d)). The rate constant inhibition (*k*_*i*_) value in plasma of quail by using AcTChI, BuTChI, PrTChI, and PSA was determined and calculated as explained in Section 2 (Table 1). *k*_*i*_ value was ranged between 122.4 × 10^−3^–567.4 × 10^−3^, 453.2 × 10^−3^–823.3 × 10^−3^, 35.8 × 10^−3^–175.6 × 10^−3^, and 345.1 × 10^−3^–976.9 × 10^−3^ min^−1^ for methomyl, parathion, malathion, and trichlorfon, respectively (Table 1). The IC_50_ values were significantly different (*P* < 0.05) between malathion with other inhibitors used in this study. The order of potency of inhibition decreased according to the rank order of malathion > methomyl > parathion > trichlorfon; the malathion ranged between (0.91–1.13 mM) higher about 4 to 5 times compared to parathion (Table 2). Esterases enzyme activity was found to be more sensitive to inhibition by parathion compared to other pesticides compounds used in this study (Table 2).

### 3.5. *In Vivo* Exposure to ChE Inhibitors

**Figure 11:**
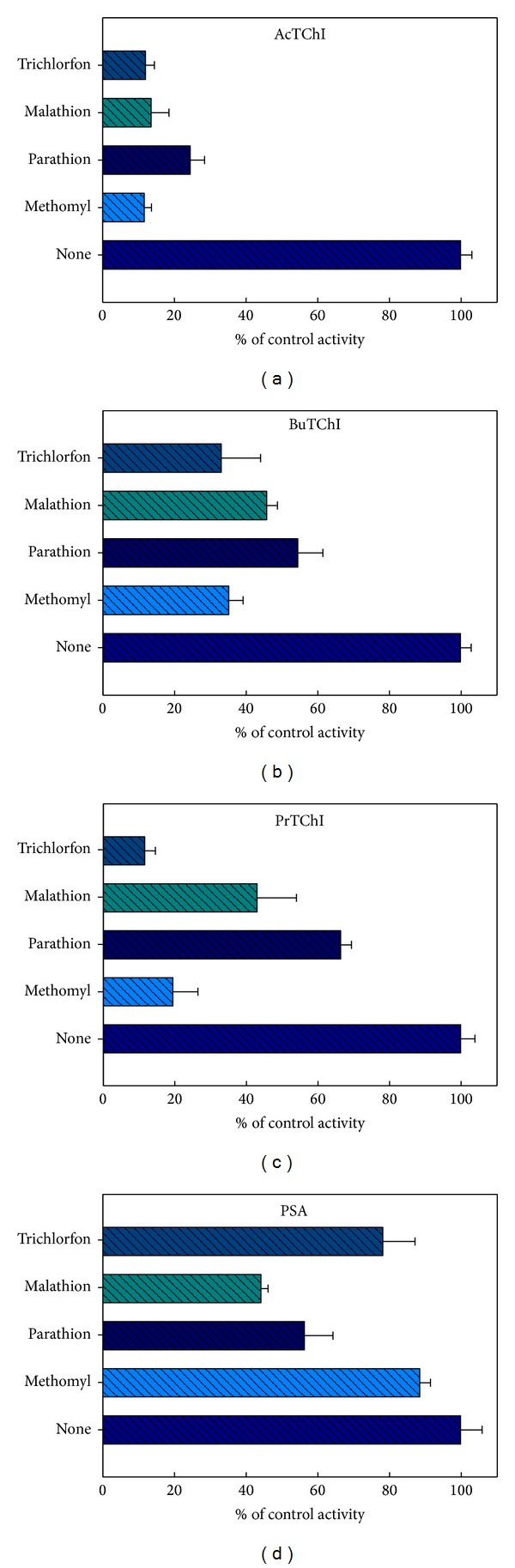
The effect of 48 h *in vivo* exposure to methomyl 12.1 mg/kg, malathion 200 mg/kg, parathion 3 mg/kg, and trichlorfon 11 mg/kg. Results are expressed as a % of the control esterases enzyme activity. Results are expressed as a % of the control esterases enzyme activity. Values in the figure are the mean ± SE obtained from nonlinear regression analysis of three individuals of blood plasma sample for quail and each experiment is performed in duplicate.

**Figure 12:**
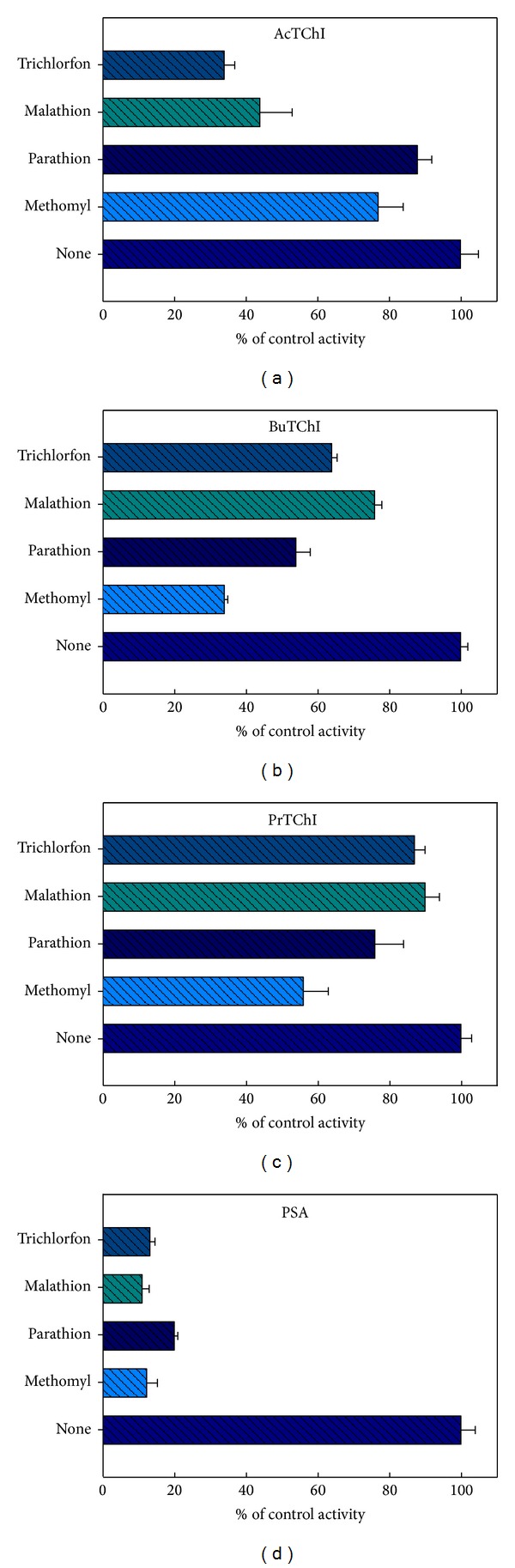
The effect of 48 h *in vivo* exposure to methomyl 12.1 mg/kg, malathion 200 mg/kg, parathion 3 mg/kg, and trichlorfon 11 mg/kg. Results are expressed as a % of the control esterases enzyme activity. Values in the figure are the mean ± SE obtained from nonlinear regression analysis of three individuals of liver homogenate quail and each experiment is performed in duplicate.

The effects of methomyl, parathion, malathion, and trichlorfon were then measured *in vivo*. Initially, quails (*n* = 15) were exposed orally to a single sublethal dose of each compound over 48 h and the CbE and ChE enzyme activities measured as described in Section 2 (Figure 11). The degree of inhibition caused by these exposure concentrations was larger for ChE than for CbE (*P* < 0.05). When expressed as % inhibition compared with the control, methomyl, parathion, malathion, and trichlorphon inhibited esterases enzyme activity in plasma by 11.8, 24.6, 13.7, and 12.1%, respectively, when used AcTChI as substrate (Figure 11(a)), while 35.3, 54.6, 45.9, and 33.2%, respectively, when used BuTChI as substrate (Figure 11(b)). PrTChI when expressed with methomyl, parathion, malathion, and trichlorphon also inhibited ChE enzyme activity in plasma by 19.6, 66.5, 43.1, and 11.7%, respectively, when used PrTChI as substrate (Figure 11(c)), while 88.6, 56.4, 44.3, and 78.3%, respectively, when used PSA as substrate (Figure 11(d)). Whilst when stated as % inhibition compared with the control, methomyl, parathion, malathion, and trichlorphon inhibited esterases enzyme activity in liver by 77.3, 88.2, 44.1, and 34.4%, respectively, when used AcTChI as substrate (Figure 12(a)), while 34.1, 54.3, 76.2, and 64.1%, respectively, when used BuTChI as substrate (Figure 12(b)). PrTChI when expressed with methomyl, parathion, malathion, and trichlorphon also inhibited ChE enzyme activity in liver by 56.7, 76.1, 90.1, and 87.3%, respectively, when used PrTChI as substrate (Figure 12(c)), while 12.3, 20.1, 11.2, and 13.3, respectively, when used PSA as substrate (Figure 12(d)). To conclude, the mean differences in the individual samples for plasma and liver homogenates between two enzymes inhibited with CB and OP pesticide compounds are plotted by Bland and Altman plot and showed clinically important, due to the mean being found lesser than ±1.96 (Figure 9(b)).

## 4. Discussion

### 4.1. Distribution and Substrate Preference of Enzyme Activity

In the last decade, the use of CbE and ChE as biomarkers for ecological monitoring has largely been supported because of their exclusive contribution to determine the toxicity of a mixture of the CB and OP pesticides, although each contaminant may be found in the habitat below the law threshold [27]. The choice of a common endpoint for the recognition of CbE or ChE enzyme activity was made to simplify both reagent preparation and recognition, the only difference between the methods being the nature of the substrate analogue. The ChE activities obtained using the BuTChI for quail blood plasma (Figure 1) are lower than those found by Dieter and Ludke [12]. However, these authors used a brain as a tissue instead of blood plasma. This factor may explain the apparent difference in activity. The added advantage of using blood plasma is the opportunity to employ repeated, nondestructive sampling. For reasons of simplicity in sample preparation, only blood plasma and liver homogenate samples rather than the other tissues of individual quail were considered.

### 4.2. Characterisation of Enzyme Activity

The differentiation of these enzyme activities in many birds models remains largely unexplained and the vertebrate classification system may be inappropriate to use [28, 29]. Bearing this in mind, the results presented in this study verify the existence of one predominant enzyme activity, classifiable as a ChE on the basis of its substrate preference, pH optimum, and high dose substrate inhibition in blood plasma of quail [30]. The level of enzyme activity was comparatively low, a characteristic described for ChE in plasma [31]. The apparent *K*_*m*_ of 0.394 mM for BuTChI calculated for this unpurified preparation is in the same order of magnitude as values described for ChE purified from the quail [32]. The hydrolysis of other substrate analogues (e.g., BuTChI), although at considerably lower levels, is in agreement with previous study of ChE activity of quail, only one of which could be classified as a ChE based on its substrate preference and susceptibility to inhibitors [33]. Therefore, interpretation of ChE enzyme activity determinations in quail plasma may be made on the basis that a single predominant toxicological form is present. Many different substrate analogues have been used in this paper for the determination of CbE activity that naturally exhibits a level of promiscuity in their substrate reactions. PSA was chosen as a substrate for monitoring CbE enzyme activity in this work because of its preferential hydrolysis (Figures 1 and 2). The CbE enzyme activity as measured by this method was higher in liver homogenate than in plasma in agreement with previous reports [34]. High levels of esterases enzyme activity have been described in the brain [35] of quail; although for the purposes of this work, the exact location of individual CbE was not measured. Although the predominant enzyme activity in the liver preparation was CbE, some ChE enzyme activity was also present displaying an order of thiocholine substrate preference of AcTChI, BuTChI, or PrTChI (Figure 3). An alternative conclusion that a single broad-spectrum enzyme activity was present, able to hydrolyse each derivative, cannot be ruled out but is less likely. The CbE is in general specific for simple carboxylic esters and lack the anionic binding site considered necessary for interaction with ChE derivatives [36]. The linear regression found of means esterase enzyme activities between plasma reaction rate (nmol min^−1^ mL^−1^) and sample volume (*μ*L) wAS directly proportional (Figures 5(a)–5(d)), in addition tended to be very high in case of AcTChI activity (*R* ^2^ = 0.98, *P* = 0.0012; Figure 5(a)), and was in all cases found that esterase enzyme activities between liver homogenate reaction rate (nmol min^−1^ g^−1^) and sample volume were (*μ*L) directly proportional (Figures 8(a)–8(d)) and tended to be very high in case of PSA activity (*R* ^2^ = 0.99, *P* = 0.0022; Figure 8(d)).

### 4.3. Clarification of Combined Esterases Enzyme Activity Determinations

Our study has presented the interpretation and characterisation of the relative sensitivities of the CbE and ChE enzyme activities to CB and OP pesticides with *in vitro* incubation confirmed the sensitivity of CbE and ChE enzyme activities to the exposure-dependent effects of selected pesticides used in this study. Generally, CbE is 10 times more sensitive than ChE to inhibition of CB and OP compounds in adult rats [37]. Sensitivity of the enzyme activities was comparable with CbE slightly more sensitive to methomyl and parathion than malathion and trichlorfon (Figures 10(a)–10(d)). Kinetic studies show that the inhibition seen at high concentrations of the malathion and trichlorfon may be due to the presence of contaminants in their preparation (Figures 10(c) and 10(d)). This is in agreement with the findings of Garcia-Repetto et al. [38]. CbE enzyme activity was insensitive to the CB methomyl except at high concentrations (Figure 10(a)). Similar patterns of decreases were also evident following *in vivo* exposures (Figure 11(d)). Both CbE and ChE activities indicated sensitivity *in vitro* to the concentrations of CB and OP compounds in the same order of magnitude although the % inhibition of ChE enzyme activity was consistently larger (Figures 10(a)–10(d)). Another important measure of ChE enzyme activity is to identify *in vivo* exposure to CB or OP compounds with statistical significance. Regardless of the similar *in vitro* sensitivities of the enzyme activities, the % inhibition of CbE enzyme activity was minor and was significantly inhibited (*P* < 0.05) only by higher amounts of CB and OP compounds [39]. Acceptable means of assessing behavioural changes in quail would no doubt be of benefit in interpretation of this species of data, as would further insight into the biological roles of the enzyme activities [40, 41]. Although the ChE enzyme activity explained in this study has been defined as such based on substrate preference and inhibitor sensitivity, there is no indication other than this that it is involved in neuromuscular enzyme activity [42, 43]. The observation of a high percentage of CB and OP compounds resistant CbE enzyme activity is striking and has been described by others birds [44] and vertebrate studies [15]. The inhibition of enzyme activity seen in blood plasma sample with *in vivo* exposure provides a net reflection of sensitivity of birds in terms of approval, detoxification, and biotransformation patterns. Equally, the relative insensitivity of enzyme activity to ecologically relevant CB and OP compounds concentrations may make it hard to accurately measure minor variations in enzyme activity against a fluctuating background of interindividual enzyme activities. Regarding distinguishing inhibitor efficiency, it is very accepted to determine IC_50_. Parathion showed higher susceptibility than methomyl, malathion, and trichlorfon (Table 2). This reason may be due to the constitution and sensitivity esterases enzyme activity in plasma that may preferentially bind parathion, thereby protecting esterases enzyme activity, resulting in greater tolerance to parathion. This agrees with the finding of Brown [45], who reported that parathion in invertebrate was more potent than methylparaoxon.

## 5. Conclusions

In conclusion, this is the first paper characterising the enzymological effects of these compounds in blood plasma and liver homogenate of quail. The interpretation of inhibition and enzyme activity exposure concentration dependent can be completed on the basis that one predominant toxicological form of the carboxylase enzyme activity is present. The determination of esterases enzyme activity in quail blood plasma using the described methodology provides a rapid, relatively low-cost and dependable means of nondestructively assessing the exposure of quail to CB and OP compounds. Measurement of esterase enzyme can be achieved with spending minimum time and with the need for just one extra reagent. Nevertheless, the clarification of CbE must take into account the large percentage of CB and OP compounds resistant enzyme activity apparent using the method explained. Whereas these assays provide a valuable means of assessing the exposure of quail to compounds, interpretation of the toxicological significance of exposure should be made with multilevel indicators and, in conjunction of toxicity, a good illustration for this is biological and behavioural changes of evidence of neurotoxic damage. Finally, results obtained from this paper also confirm the suitability of tissues for Ellman esterase enzyme determinations in quail samples.

## Figures and Tables

**Table 1:** Rate constant inhibition (*k*_*i*_) × 10^−3^ min^−1^ characterization by using different esterase enzyme substrates (AcTChI, BuTChI, PrTChI, and PSA) for blood plasma sample of quail inhibited by CB and OP.

Pesticides	AcTChI	BuTChI	PrTChI	PSA
Methomyl	122.4 ± 4.5	456.0 ± 3.2	567.4 ± 8.7	345.3 ± 12.4
Parathion	734.6 ± 1.8	823.3 ± 3.5	789.0 ± 15.6	453.2 ± 5.6
Malathion	35.8 ± 9.7*	65.2 ± 6.0*	117 ± 12.0*	175.6 ± 16.0*
Trichlorfon	668.4 ± 4.3	341.1 ± 11.2	645.7 ± 6.7	976.9 ± 8.9

Values in the table are the mean ± SE of three individual quails. The measured data resulted in a calculation over different times (1–60 min) resulting in an inhibition of 83–99% of control activity by inhibition with 4 mM methomyl and 2 mM parathion, malathion, and trichlorfon. Then results were fitted with a single exponential decay using SigmaPlot 11. *Significant difference (analysis of variance (ANOVA), *P* < 0.05) between malathion with other pesticide compounds (methomyl, parathion and trichlorfon).

**Table 2:** Half maximal inhibitory concentration (IC_50_; mM) characterization by using different esterase enzyme substrates (AcTChI, BuTChI, PrTChI, and PSA) for blood plasma sample of quail inhibited by CB and OP compounds.

Pesticides	AcTChI	BuTChI	PrTChI	PSA
Methomyl	0.71 ± 0.05	0.92 ± 0.02	0.86 ± 0.07	0.61 ± 0.02
Parathion	0.12 ± 0.02	0.24 ± 0.05	0.66 ± 0.06	0.22 ± 0.06
Malathion	0.93 ± 0.2*	1.11 ± 0.9*	1.13 ± 0.2*	0.91 ± 0.4*
Trichlorfon	0.31 ± 0.03	0.26 ± 0.08	0.7 ± 0.01	0.51 ± 0.09

Values in the table are the mean ± SE of three individual quails. For the measurement of IC_50_, esterase enzyme was inhibited for 30 min at room temperature 25°C with either 0.25–4 mM CB or 0.125–2 mM OP compounds. Then results were fitted with an exponential decay using SigmaPlot 11. *Significant difference (analysis of variance (ANOVA), *P* < 0.05) between malathion with other pesticide compounds (methomyl, parathion, and trichlorfon).

## Abbreviations

ANOVA: Analysis of variance
AcTChI: Acetylthiocholine iodide
BuTChI: Butyrylthiocholine iodide
CB: Carbamate
CbE: Carboxylesterase
CV: Coefficient of variance
ChE: Cholinesterase
cm: Centimetre
DTNB: 5,5′-Dithiobis(2-nitrobenzoic acid)
EC: Enzyme commission
g: Specific gravity
g: Gram
IC_50_: Half maximal inhibitory concentrations
*K*_*m*_: Michaelis-Menten constant
*k*_*i*_: Rate constant for inhibition
LD_50_: Median lethal dose
mm: Millimeter
mM: Millimolar
nm: Nanometer
nmol: Nanomole
OP: Organophosphate
PrTChI: Propionylthiocholine iodide
PSA: S-Phenyl thioacetate
rpm: Revolutions per minute
SE: Standard error
TNB: 5-Thio-2-nitrobenzoic acid
*μ*mol: Micromole
*μ*M: Micromolar
v/v: Volume/volume
*V*_max_: Maximum reaction velocity at a given substrate concentration
w/v: Weight/volume.

## References

[1] Fossi MC, Leonzio C, Massi A, Lari L, Casini S (1992). Serum esterase inhibition in birds: a nondestructive biomarker to assess organophosphorus and carbamate contamination. *Archives of Environmental Contamination and Toxicology*.

[2] Askar K, Kudi AC, Moody AJ (2010). Comparison of two storage methods for the analysis of cholinesterase activities in food animals. *Enzyme Research*.

[3] Nigg HN, Knaak JB (2000). Blood cholinesterases as human biomarkers of organophosphorus pesticide exposure. *Reviews of Environmental Contamination and Toxicology*.

[4] Wilson BW, Philip W (2005). Cholinesterase inhibition. *Encyclopaedia of Toxicology*.

[5] Fukuto TR (1990). Mechanism of action of organophosphorus and carbamate insecticides. *Environmental Health Perspectives*.

[6] Moore DR, Teed RS (2013). Risks of carbamate and organophosphate pesticide mixtures to salmon in the Pacific Northwest. *Integrated Environmental Assessment and Management*.

[7] Askar KA, Kudi AC, Moody AJ (2011). Comparative analysis of cholinesterase activities in food animals using modified Ellman and Michel assays. *Canadian Journal of Veterinary Research*.

[8] Sandrini JZ, Rola RC, Lopes FM (2013). Effects of glyphosate on cholinesterase activity of the mussel Perna perna and the fish Danio rerio and Jenynsia multidentata: *In Vitro* studies. *Aquatic Toxicology*.

[9] Silveyra MX, García-Ayllón MS, de Barreda EG (2012). Altered expression of brain acetylcholinesterase in FTDP-17 human tau transgenic mice. *Neurobiology of Aging*.

[10] Low V, Chen C, Lee H, Lim P, Leong C, Sofian-Azirun M (2013). Current susceptibility status of malaysian culex quinquefasciatus (Diptera: Culicidae) against DDT, propoxur, malathion, and permethrin. *Journal of Medical Entomology*.

[11] Stewart DJ, Inaba T, Tang BK, Kalow W (1977). Hydrolysis of cocaine in human plasma by cholinesterase. *Life Sciences*.

[12] Dieter MP, Ludke JL (1975). Studies on combined effects of organophosphates and heavy metals in birds. I. Plasma and brain cholinesterase in Coturnix quail fed methyl mercury and orally dosed with parathion. *Bulletin of Environmental Contamination and Toxicology*.

[13] Hosokawa M, Maki T, Satoh T (1990). Characterization of molecular species of liver microsomal carboxylesterases of several animal species and humans. *Archives of Biochemistry and Biophysics*.

[14] Wheelock CE, Shan G, Ottea J (2005). Overview of carboxylesterases and their role in the metabolism of insecticides. *Journal of Pesticide Science*.

[15] Sogorb MA, Vilanova E (2002). Enzymes involved in the detoxification of organophosphorus, carbamate and pyrethroid insecticides through hydrolysis. *Toxicology Letters*.

[16] Correll L, Ehrich M (1987). Comparative sensitivities of avian neural esterases to *In Vitro* inhibition by organophosphorus compounds. *Toxicology Letters*.

[17] Fairbrother A, Marden BT, Bennett JK, Hooper MJ, Minneau P (1991). Methods used in determination of cholinesterase activity. *Chemicals in Agriculture*.

[18] Ellman GL, Courtney KD, Andres V, Featherstone RM (1961). A new and rapid colorimetric determination of acetylcholinesterase activity. *Biochemical Pharmacology*.

[19] Nilin J, Monteiro M, Domingues I, Loureiro S, Costa-Lotufo LV, Soares AMVM (2012). Bivalve esterases as biomarker: identification and characterization in European cockles (*Cerastoderma edule*). *Bulletin of Environmental Contamination and Toxicology*.

[20] Trimm JL, Salama G, Abramson JJ (1986). Sulfhydryl oxidation induces rapid calcium release from sarcoplasmic reticulum vesicles. *Journal of Biological Chemistry*.

[21] Wever R, Kast WM, Kasinoedin JH, Boelens R (1982). The peroxidation of thiocyanate catalysed by myeloperoxidase and lactoperoxidase. *Biochimica et Biophysica Acta*.

[22] U.S. Environmental Protection Agency (1987). *Health Advisory Summary: Methomyl*.

[23] Meister RT (1992). *Farm Chemicals Handbook*.

[24] Griffin LLC (1999). Material safety data sheet. *Ecological Information*.

[25] Hill EF, Camardese MB (1986). Lethal dietary toxicities of environmental contaminants to coturnix.

[26] Dewitte K, Fierens C, Stöckl D, Thienpont LM (2002). Application of the Bland-Altman plot for interpretation of method-comparison studies: a critical investigation of its practice. *Clinical Chemistry*.

[27] Barr DB, Needham LL (2002). Analytical methods for biological monitoring of exposure to pesticides: a review. *Journal of Chromatography B*.

[28] Thompson HM, Walker CH, Hardy AR (1991). Changes in activity of avian serum esterases following exposure to organophosphorus insecticides. *Archives of Environmental Contamination and Toxicology*.

[29] Askar KA, Kudi AC (2012). *In Vitro* kinetic characterization of inhibition of acetylcholinesterase by organophosphate and carbamate compounds in food animals. *Toxicological and Environmental Chemistry*.

[30] Hill EF (1989). Sex and storage affect cholinesterase activity in blood plasma of Japanese quail. *Journal of wildlife diseases*.

[31] Dieter MP (1974). Plasma enzyme activities in Coturnix quail fed graded doses of DDE, polychlorinated biphenyl, malathion and mercuric chloride. *Toxicology and Applied Pharmacology*.

[32] Boily MH, Ndayibagira A, Spear PA (2003). Retinoid metabolism (LRAT, REH) in the yolk-sac membrane of Japanese quail eggs and effects of mono-ortho-PCBs. *Comparative Biochemistry and Physiology C*.

[33] Ferrand R, Sine JP, Colas B (1993). Butyrylcholinesterase from intestinal epithelial cells of quail, chick and duck: a comparative study during development. *Comparative Biochemistry and Physiology B*.

[34] Cohen SD, Murphy SD (1970). Comparative potentiation of malathion by triorthotolyl phosphate in four classes of vertebrates. *Toxicology and Applied Pharmacology*.

[35] Westlake GE, Bunyan PJ, Martin AD, Stanley PI, Steed LC (1981). Organophosphate poisoning. Effects of selected organophosphate pesticides on plasma enzymes and brain esterases of Japanese quail (*Coturnix coturnix japonica*). *Journal of Agricultural and Food Chemistry*.

[36] Wogram J, Sturm A, Segner H, Liess M (2001). Effects of parathion on acetylcholinesterase, butyrylcholinesterase, and carboxylesterase in three-spined stickleback (Gasterosteus aculeatus) following short-term exposure. *Environmental Toxicology and Chemistry*.

[37] Chanda SM, Mortensen SR, Moser VC, Padilla S (1997). Tissue-specific effects of chlorpyrifos on carboxylesterase and cholinesterase activity in adult rats: an *In Vitro* and in vivo comparison. *Toxicological Sciences*.

[38] Garcia-Repetto R, Martinez D, Repetto M (1995). Malathion and dichlorvos toxicokinetics after the oral administration of malathion and trichlorfon. *Veterinary and Human Toxicology*.

[39] Kassa J, Cabal J (1999). A comparison of the efficacy of acetylcholinesterase reactivators against cyclohexyl methylphosphonofluoridate (GF agent) by *In Vitro* and in vivo methods. *Pharmacology and Toxicology*.

[40] Adkins EK, Alder NT (1972). Hormonal control of behavior in the Japanese quail. *Journal of Comparative and Physiological Psychology*.

[41] Panzica GC, Viglietti-Panzica C, Balthazart J (1996). The sexually dimorphic medial preoptic nucleus of quail: a key brain area mediating steroid action on male sexual behavior. *Frontiers in Neuroendocrinology*.

[42] Skladal P, Mascini M (1992). Sensitive detection of pesticides using amperometric sensors based on cobalt phthalocyanine-modified composite electrodes and immobilized cholinesterases. *Biosensors and Bioelectronics*.

[43] Fournier D, Mutero A (1994). Modification of acetylcholinesterase as a mechanism of resistance to insecticides. *Comparative Biochemistry and Physiology C*.

[44] Ehrich M, Jortner BS, Taylor D, Dunnington EA, Siegel PB (1993). Differences between genetic stocks of chickens in response to acute and delayed effects of an organophosphorus compound. *Journal of Toxicology and Environmental Health*.

[45] Brown TM (1992). Selective inhibitors of methyl parathion-resistant acetylcholinesterase from Heliothis virescens. *Pesticide Biochemistry and Physiology*.

